# Influence of Histone Acetylation on Chromatin 3D Organization in *Drosophila* S2 Cells

**DOI:** 10.3390/ijms27198638

**Published:** 2026-09-27

**Authors:** Anna D. Kononkova, Sergey V. Ulianov, Eugenia A. Tiukacheva, Elena A. Mikhaleva, Semen A. Doronin, Victoria A. Kobets, Ekaterina E. Khrameeva, Sergey V. Razin, Yuri Y. Shevelyov

**Affiliations:** 1 Center for Bio- and Medical Technologies, The Territory of the Skolkovo Innovation Center, Moscow 121205, Russia; kononkova@gmail.com (A.D.K.); victory.kobets@gmail.com (V.A.K.); 2 Department of Cellular Genomics, Institute of Gene Biology, Russian Academy of Sciences, Moscow 119334, Russia; sergey.v.ulyanov@gmail.com (S.V.U.); teja98@mail.ru (E.A.T.); sergey.v.razin@inbox.ru (S.V.R.); 3 Faculty of Biology, M.V. Lomonosov Moscow State University, Moscow 119991, Russia; 4 Laboratory of Molecular Genetics of Animals, National Research Centre “Kurchatov Institute”, Moscow 123182, Russia; mikhalevaelena.img@yandex.ru; 5 Laboratory of Analysis of Gene Regulation, National Research Centre “Kurchatov Institute”, Moscow 123182, Russia; semdornot@gmail.com; 6 Research and Development Department, Medical Company “Genomed”, Moscow 115419, Russia

**Keywords:** genome architecture, histone acetylation, trichostatin A, curcumin, HDAC1, topologically associating domains, higher-order chromatin organization, *Drosophila*

## Abstract

Alterations in histone acetylation are frequently detected in various cancers and neurological diseases. Histone acetylation is known to disrupt both intra- and inter-nucleosomal interactions, but its influence on higher-order chromatin organization has been studied to a lesser extent. In this study, we changed global levels of histone acetylation in *Drosophila* S2 cells by treatment with trichostatin A (TSA; an inhibitor of histone deacetylases) or with curcumin (an inhibitor of histone acetyltransferases and histone deacetylases). We also performed a knockdown of the main histone deacetylase HDAC1/Rpd3 in S2 cells. According to ChIP-seq analysis, histone H4 pan-acetylation was mostly increased in the active chromatin regions and decreased in the inactive regions after TSA treatment, whereas after curcumin treatment, it changed in the opposite direction. Changes in the level of histone acetylation affect the packaging density of topologically associating domains (TADs), which are the units of higher-order chromatin organization. Importantly, increased histone acetylation leads to decreased packaging density of TADs and vice versa. Moreover, the increased histone acetylation within active TADs enhances their spatial segregation from the inactive ones. Our findings highlight the key role of histone acetylation in determining the 3D organization of chromatin domains, supporting the idea that the separation of active and inactive TADs in *Drosophila*, at least in part, is mediated by histone acetylation. These results may help to understand how agents such as histone deacetylase inhibitors exert their therapeutic effects in certain cancers and other human diseases.

## 1. Introduction

The nucleosomal and higher-order chromatin organization enables the genome to fit into the eukaryotic nucleus, but dense packaging of DNA interferes with transcription, replication, and recombination. Histone acetylation is known to facilitate all these processes [1]. In addition, acetylated histones represent binding sites for bromodomain-containing proteins, some of which promote transcription [2]. Not surprisingly, changes in histone acetylation are associated with various types of human diseases, including cancer and neurological disorders [3].

It is well known that housekeeping genes possess acetylated chromatin in all cell types, while tissue-specific genes may have either acetylated or deacetylated chromatin depending on whether gene expression is turned on or off. Chromatin transition from a transcriptionally inactive to an active state is initiated by the binding of transcriptional activators, which further recruit chromatin remodeling complexes and acetyltransferases, locally increasing histone acetylation within a chromatin domain [4]. Initiation of gene expression is usually accompanied by relocalization of the activated loci from the nuclear lamina (NL) to the nuclear interior [5].

With the advent of high-throughput chromosome conformation capture (Hi-C) technology [6], it became clear that chromosomes of various multicellular organisms are partitioned into contact domains of different nature [7,8,9,10,11]. In mammals, cohesin-mediated loop extrusion, which is stopped by CTCF roadblocks bound to convergent CTCF-binding sites, is the main process that determines the formation and demarcation of topologically associating domains (TADs) [12,13,14]. However, after depletion of CTCF or components of the cohesin complex, a different pattern of contact domains appears, which corresponds to the alternation of active and inactive chromatin domains [15,16,17], thus indicating that two distinct mechanisms govern TAD formation in mammals [18]. In *Drosophila*, CTCF is not enriched at TAD boundaries [19,20,21] and is not able to execute cohesin-blocking functions due to the loss of a short linker sequence consisting of basic amino acid residues adjoining Zn-finger domains at the N-terminus [22]. As a result, TADs in *Drosophila* are mainly collinear with active and inactive chromatin domains [10,11,18,19,23].

Biophysical principles of phase separation were invoked to explain the spatial segregation of active and inactive chromatin into two compartments [24,25,26]. Seminal in vitro experiments indicate that the non-acetylated histone H4 tail of one nucleosome is able to bind to the acidic patch interface formed by the histone H2A/H2B dimer of another nucleosome, thus leading to chromatin fiber compactization, whereas histone acetylation prevents inter-nucleosomal interactions [27,28,29,30,31]. Based on these observations, we have suggested that the ability of non-acetylated nucleosomes to aggregate with each other and the lack of this ability in acetylated nucleosomal arrays may be the main driving force that spatially separates active and inactive TADs in *Drosophila* [19]. Recent in vitro reconstitution of extended (~20 kb) acetylated nucleosome arrays has shown that histone H4 acetylation indeed causes decompactization of chromatin domains [32]. Moreover, alternating acetylated and non-acetylated nucleosomal arrays were shown to be sufficient for spatial separation of active and inactive chromatin [32], thereby confirming the proposed mechanism of TAD formation in *Drosophila* [19]. In addition to these basic properties of nucleosomes, the heterochromatin protein HP1 and the linker histone H1 were found to enhance the formation of phase separation droplets consisting of non-acetylated nucleosomes [33,34,35], while proteins containing bromodomains, on the contrary, stimulate the formation of droplets consisting of acetylated nucleosomes [35].

Apart from in vitro experiments, several in vivo studies examined the effects of acetylation on chromatin compaction. Treatment of HeLa cells with trichostatin A (TSA) was found to cause chromatin decondensation [36]. Recent super-resolution microscopy studies have shown that acetylated chromatin domains have lower packaging density than non-acetylated ones [37,38,39]. Furthermore, the level of acetylation of chromatin domains negatively correlates with their packaging density [40].

Despite all these in vitro and in vivo studies, it remains unclear how changes in histone acetylation affect the compaction of TADs. In this work, we address this question using the *Drosophila* S2 cell model.

## 2. Results

### 2.1. Treatment of S2 Cells with TSA or Curcumin Notably Affects Global Levels of Histone Acetylation Within Active and Inactive Chromatin Domains

To change the level of histone acetylation in *Drosophila* S2 cells, we treated cells with the histone deacetylase inhibitor TSA [41], or with curcumin. TSA inhibits class I and class II histone deacetylases by chelating the zinc ion located within the enzymes’ active-site pocket [42]. Although curcumin was reported to inhibit the activity of histone acetyltransferase CBP/p300 [43,44] (likely via its chemical modification by alkylation followed by degradation [45]) as well as that of histone deacetylases [46,47,48] (through a mechanism similar to that of TSA [49,50]), treatment of S2 cells with curcumin clearly reduced the acetylation levels of histones H3 and H4 (Figure 1A). By contrast, treatment of S2 cells with TSA increased the acetylation levels of both histones (Figure 1A).

To analyze changes in histone acetylation genome-wide, we performed chromatin immunoprecipitation sequencing (ChIP-seq) using antibodies against pan-acetylated histone H4 (H4panAc)—which recognize histone H4 acetylated at Lys5, Lys8, Lys12, and Lys16—in control S2 cells (treated with DMSO) and in cells treated with TSA or curcumin. Visual examination of acetylation profiles indicates that, after TSA treatment, histone acetylation was mostly increased in active chromatin regions and decreased in inactive regions, whereas after curcumin treatment, histone acetylation changed in the opposite direction (Figure 1B and Figure 2A). Bioinformatics analysis confirmed that, after TSA treatment, histone acetylation was increased within active chromatin regions and decreased within inactive ones (Figure 1C), while curcumin treatment had the opposite effect.

### 2.2. Global Changes in Histone Acetylation Affect the Packaging Density of Super-TADs

To trace alterations in the spatial organization of chromatin in response to acetylation changes, we first performed a pilot, low-resolution Hi-C analysis (using the HindIII restriction enzyme and 20-kb bins) in control (DMSO-treated) and TSA- or curcumin-treated S2 cells. It was previously shown that the so-called super-TADs, which were identified by low-resolution Hi-C in S2 cells [19], actually correspond to clusters of smaller TADs revealed by Hi-C at sub-kb resolution [21]. This subdivision was particularly notable for super-TADs and inter-super-TADs consisting of active chromatin [21].

By applying *Armatus* software [52] to our low-resolution Hi-C data, we thus identified super-TADs in TSA-treated, curcumin-treated, and control cells (Figure 2B; Supplementary Table S1). The majority of super-TADs (710 for TSA and 516 for curcumin) had nearly the same boundary positions in control and treated cells (Figure 2B). We then divided super-TADs into four groups (A, B, C, D) depending on their coverage by active (types 1–5 according to [51]) and inactive (types 6–9 according to [51]) chromatin types (Supplementary Table S2). As a result, group A super-TADs contained the largest proportion of active chromatin, while group D super-TADs contained the lowest proportion (see Materials and Methods for details). Of note, for this and further analyses, we considered only autosomal super-TADs (or TADs), since the X-chromosome has a distinct chromatin type established by the dosage compensation complex operating in S2 cells [53]. Importantly, according to our ChIP-seq analysis, TSA treatment of cells led to increased histone acetylation in group A super-TADs and decreased histone acetylation in group D super-TADs, as compared to control cells (Figure 2C, left panel; Supplementary Table S2). Conversely, after curcumin treatment, group D super-TADs became more acetylated, whereas group A super-TADs became less acetylated (Figure 2C, right panel; Supplementary Table S2).

We reasoned that if we compare the average contact frequency (ACF) of a particular super-TAD (which has the same size, genomic position, and predominant chromatin type in control and treated cells), then changes in the ACF value would reflect changes in the packaging density of this super-TAD. This approach has shown that, after TSA treatment, the median ACF value (and thus the packaging density) of the most active super-TADs (group A) was diminished, while that of the most inactive super-TADs (group D) was increased (Figure 2D, left panel; Supplementary Table S2). Conversely, after curcumin treatment, the median ACF value of group A super-TADs increased, while it significantly decreased in group D super-TADs (Figure 2D, right panel; Supplementary Table S2). Therefore, we observed an inverse correlation between changes in histone acetylation within super-TADs and changes in their packaging density. In other words, a gain in histone acetylation within a super-TAD results in its decompaction.

Next, we examined what happens to the distant chromatin contacts within active (A) and inactive (B) compartments [6] upon TSA or curcumin treatment. Saddle plots demonstrate that, upon TSA treatment, distant contacts are enhanced in the A compartment and are mostly weakened in the B compartment, whereas upon curcumin treatment the changes are in the opposite direction (Figure 2E). Thus, decompaction of active super-TADs that occurs upon TSA treatment results in an enhanced contact frequency between distant TADs within the A compartment.

### 2.3. Global Changes in Histone Acetylation Affect the Packaging Density of TADs

Then, we analyzed the effect of the increased histone acetylation on the packaging density of TADs. Using RNA interference in S2 cells, we performed a knockdown of the gene encoding the main histone deacetylase HDAC1/Rpd3 (hereafter HDAC1-KD) (Figure 3A). Consistent with Western blot data from [41], which showed that increased histone H3 acetylation in S2 cells upon TSA treatment is mainly mediated by TSA inhibition of HDAC1, our results indicate that HDAC1-KD resulted in a similar increase in histone H3K9 acetylation to TSA treatment (Figure 3B). Immunostaining of S2 cells with antibodies against H3K9Ac confirmed the elevated level of histone acetylation in HDAC1-KD cells compared to control cells (Figure 3C).

Next, we carried out high-resolution Hi-C analysis (using the DpnII restriction enzyme and 2-kb bins) in control and HDAC1-KD S2 cells and identified many more TADs than with low-resolution Hi-C (Supplementary Table S1). The identified TADs mainly coincided either with active or with inactive chromatin domains (Figure 3D). After dividing these TADs into four groups depending on chromatin type, we calculated changes in their ACF values (Supplementary Table S2). Similar to what was observed with low-resolution Hi-C, high-resolution Hi-C showed that, upon HDAC1-KD, an increase in histone acetylation within small active TADs (from group A) resulted in a decrease in the ACF values of these TADs (i.e., their decompaction), whereas inactive TADs became more compact (Figure 3E).

Additionally, we analyzed changes in ACF values upon HDAC1-KD within A and B compartments (Figure 3F). As with TSA treatment, HDAC1-KD resulted in an increased number of distant contacts within the A compartment and a decreased number of distant contacts within the B compartment.

### 2.4. Changes in Histone Acetylation upon HDAC1-KD Strengthen Boundaries Between TADs

Finally, we analyzed whether changes in histone acetylation alter the strength of boundaries between different groups of TADs. We calculated the average insulation score [54], which characterizes the frequency of contacts across the boundaries (a lower insulation score indicates a lower frequency of contacts across the boundary, i.e., a stronger boundary). As expected, in control cells, the deepest valley in the average insulation score was observed at the boundaries that separate active (group A) and inactive (group D) TADs (Figure 4, right panel). HDAC1-KD resulted in a decreased value of the average insulation score at all types of boundaries, but most prominently at the boundaries that delimit active and inactive TADs (Figure 4). Therefore, the increased histone acetylation within active TADs particularly enhances their spatial isolation from TADs composed of inactive chromatin.

## 3. Discussion

In this study, we analyzed what happens to the compaction of TADs (or super-TADs) in response to induced or reduced histone acetylation in *Drosophila* S2 cells. First of all, we found that treatment of cells with either TSA or curcumin increases or decreases, respectively, histone acetylation in active chromatin regions, where histone acetylation was rather high in control cells (Figure 1B,C and Figure 2C). In TSA-treated mammalian cells, ChIP-seq showed that increased histone acetylation was largely confined to active chromatin domains [55,56]. These findings are consistent with the fact that histone acetyltransferases and histone deacetylases are preferentially localized to actively expressed genes [57,58]. However, both treatments change the level of histone acetylation in inactive chromatin as well, which becomes slightly less acetylated after TSA treatment or slightly more acetylated after curcumin treatment (Figure 1B,C and Figure 2C). We suggest the following explanation for this fact. Upon TSA treatment, the enhanced acetylation in active chromatin regions may reduce the level of acetyl-CoA, a substrate for acetyltransferases. This would result in slightly weaker occasional acetylation in inactive chromatin regions. On the other hand, the increased level of acetyl-CoA after curcumin treatment would result in slightly enhanced occasional acetylation in inactive chromatin regions due to the action of acetyltransferases that were not inhibited by curcumin.

Most importantly, we found that artificial enhancement of global histone acetylation causes the decompaction of TADs and super-TADs consisting of active chromatin, whereas weakening of global histone acetylation leads to a more compact state of inactive TADs and super-TADs (Figure 2D and Figure 3E). Moreover, we have shown that increased histone acetylation strengthens boundaries between TADs (Figure 4). Our results, indicating that increased histone acetylation in active TADs promotes their segregation from inactive ones, support the idea that spatial separation of active and inactive chromatin is, at least in part, mediated by histone acetylation [19,32]. These findings are in line with the in vitro analysis of chromatin fiber compaction [27,28,29,30,31,32], as well as with super-resolution microscopy data showing that acetylated chromatin domains are less densely packed than non-acetylated ones [37,38,39,40,59].

A recent study has addressed the role of histone acetylation in chromatin compaction by employing Hi-C analysis in mammalian cells [60]. The authors have shown that treatment of cells with TSA resulted in the weakening of intra-TAD interactions [60]. Since in mammals, the formation of TADs is primarily determined by cohesin-mediated loop extrusion [12,13,14] and TSA treatment affects cohesin interactions with DNA [60], it is a challenge to distinguish the influence of loop extrusion from that of inter-nucleosomal interactions on the decompaction of TADs upon TSA treatment. In this respect, *Drosophila* is a simpler system for such analysis, because in this organism, loop extrusion likely does not play a distinct role in the formation of TADs [14] and the spatial separation of TADs is mainly determined by the segregation of active and inactive chromatin domains [10,11,18,19,23]. Thus, our results on *Drosophila* complement the results in mammals [60] and demonstrate that decompaction of TADs in response to increased histone acetylation is, at least in part, mediated by disruption of inter-nucleosomal interactions. Taken together, all these data clearly indicate that there is a causal relationship between increased or decreased histone acetylation and the loosening or compactization of higher-order chromatin structures and that inter-nucleosomal interactions play a substantial role in determining these structures.

To our knowledge, this is the first analysis of changes in TAD compaction in response to altered histone acetylation in *Drosophila*. Because TADs represent fundamental units of spatial chromatin organization, within which enhancers recognize their target promoters [61], these findings may help to understand how agents such as histone deacetylase inhibitors exert their therapeutic effects in certain cancers and other human diseases.

It should be noted that our study has several main limitations. First, it was performed on the well-characterized and commonly used *Drosophila* S2 cells. However, these cells are of embryonic origin [62] and thus their chromatin is less condensed than in differentiated cells [63]. In particular, S2 cells lack lamin C [64], which could influence chromatin organization in more differentiated cell types. In future studies, it would be interesting to analyze how increased or decreased histone acetylation impacts 3D chromatin organization in differentiated cell types containing a more mature chromatin structure. Second, TSA and curcumin affect the acetylation of histones as well as of numerous non-histone proteins [65]. Some of these proteins may also influence chromatin organization. However, our HDAC1-KD experiments are more direct and specific in this respect. More problematically, histone acetylation creates bromodomain binding sites that recruit the transcription machinery and chromatin remodelers. As a result, the decompaction of active TADs that we detected upon TSA or HDAC1-KD treatment may be mediated not only by disruption of inter-nucleosomal interactions but also by the action of nucleosome-remodeling and transcription complexes. Third, the weak decompaction of active TADs upon HDAC1-KD in Figure 3E may be caused by incomplete depletion of HDAC1. Currently, highly specific HDAC inhibitors do exist [66], which may be applied to clarify this question in future studies.

## 4. Materials and Methods

### 4.1. Cell Culture Maintenance and Treatment

The *Drosophila melanogaster* S2 cell line (from the collection of the Institute of Molecular Genetics) was grown at 25 °C in Schneider’s Drosophila Medium (Gibco, Waltham, MA, USA) supplemented with 10% heat-inactivated fetal bovine serum (FBS, Gibco, Waltham, MA, USA), 50 units/mL penicillin, and 50 µg/mL streptomycin. To change the level of histone acetylation, S2 cells were treated with 50 ng/mL TSA (dissolved in dimethyl sulfoxide (DMSO); T8552, Sigma-Aldrich, St. Louis, MO, USA) or 50 µM curcumin (dissolved in DMSO; C1386, Sigma-Aldrich, St. Louis, MO, USA) for 24 h. Control cells were treated with 6 µL/mL DMSO (D2650, Sigma-Aldrich). To perform HDAC1-KD in S2 cells, dsRNA against *lacZ* (control) was prepared as previously described [67]. dsRNA against *HDAC1*/*Rpd3* was prepared in the same fashion using *Drosophila* genomic DNA as a template for PCR amplification with the 5′-GAATTAATACGACTCACTATAGGGAGAGCAGCGTTTCAATGTCGGCGAAGATT and 5′-GAATTAATACGACTCACTATAGGGAGATCATCGTTGATGGCATCCTCGGGAA primers. Treatment of cells with dsRNAs was performed over four days using a previously described protocol [68].

### 4.2. Western Blot Analysis

Proteins were extracted with 8 M urea, 0.1 M Tris-HCl, pH 7.0, 1% SDS, fractionated by SDS-PAGE (12% acrylamide gel) and transferred to a PVDF membrane (Immobilon-P, Millipore, Butlington, MA, USA). Blots were developed using alkaline phosphatase-conjugated secondary antibodies (Sigma-Aldrich, St. Louis, MO, USA) and the ImmunStar AP detection system (Bio-Rad, Hercules, CA, USA). The following antibodies were used for detection: rabbit polyclonal anti-H4panAc (1:800; Active Motif 39026, Carlsbad, CA, USA), rabbit polyclonal anti-H3K9Ac (1:1000; Abcam 10812, Cambridge, UK), rabbit polyclonal anti-histone H3 (1:2500; Abcam ab1791, Cambridge, UK), rabbit monoclonal anti-histone H4 (1:1000; Abcam ab177840, Cambridge, UK), rabbit polyclonal anti-HDAC1 (1:500; Abcam ab1767, Cambridge, UK), and mouse monoclonal anti-Actin (1:5000; Abcam ab8224, Cambridge, UK) antibodies.

### 4.3. Immunostaining of S2 Cells

S2 cells were seeded on coverslips for 30 min. The subsequent immunostaining procedure was performed as previously described [69]. As primary antibodies, we used rabbit polyclonal anti-H3K9Ac (1:500; Abcam 10812, Cambridge, UK) and mouse monoclonal anti-LamDm0 (ADL84, Developmental Studies Hybridoma Bank, Iowa City, IA, USA) antibodies. As secondary antibodies, we used Alexa Fluor 633-conjugated goat anti-rabbit IgG (Invitrogen, Waltham, MA, USA) or Alexa Fluor 488-conjugated goat anti-mouse IgG (Invitrogen, Waltham, MA, USA) antibodies.

### 4.4. ChIP-Seq Procedure

Cells were fixed with 1% formaldehyde for 10 min at room temperature. The reaction was quenched with 125 mM glycine. Cells were pelleted, washed with PBS, and lysed in the RIPA buffer for 15 min on ice. Chromatin was sonicated to 100–500 bp, concentrated in Amicon 30K filters (Millipore, Butlington, MA, USA), and diluted with RIPA buffer to 500 μL; 10% was taken as an input control. Chromatin was incubated with antibodies against pan-acetylated histone H4 (Active Motif 39026) overnight at 4 °C with rotation. The next day, chromatin was incubated with pre-washed A/G ChIP beads (Thermo Scientific, Waltham, MA, USA) for 6 h at 4 °C with rotation. After incubation, samples on the beads were washed and resuspended in 100 μL of PBS. To each sample, 4 μL of 20% SDS and 100 μg of Proteinase K were added. All samples and controls were incubated at 65 °C overnight with constant shaking. DNA was extracted with a phenol:chloroform solution (1:1) and ethanol-precipitated with tRNA and glycogen as co-precipitators. Purified DNA was dissolved in 50 μL of 10 mM Tris-HCl, pH 8.0. The DNA ends were repaired by adding 62.5 μL H_2_O, 14 μL 10 × T4 DNA ligase buffer, 3.5 μL 10 mM dNTP mix, 5 μL of T4 DNA polymerase (3 U/μL, New England Biolabs, Ipswich, MA, USA), 5 μL of T4 polynucleotide kinase (10 U/μL, New England Biolabs), and 1 μL of Klenow DNA polymerase (5 U/μL, New England Biolabs), during incubation at 20 °C for 30 min. DNA was purified with Agencourt AMPure XP beads (Beckman Coulter, Brea, CA, USA) and eluted with 50 μL of 10 mM Tris-HCl (pH 8.0). To perform an A-tailing reaction, DNA samples were supplemented with 6 μL 10 × NEBuffer 2, 1.2 μL 10 mM dATP, 1 μL H_2_O, and 3.6 μL Klenow (exo-) fragment (5 U/μL, New England Biolabs, Ipswich, MA, USA). The reactions were performed for 30 min at 37 °C, and the enzyme was then heat-inactivated by incubation at 65 °C for 20 min. DNA was purified using Agencourt AMPure XP beads (Beckman Coulter, Brea, CA, USA). Adapter ligation was performed in a mixture containing 41.5 μL DNA, 5 μL 10 × T4 DNA ligase buffer, 2.5 μL Illumina TruSeq adapters, and 1 μL T4 DNA ligase (5 U/μL, Thermo Scientific, Waltham, MA, USA) at 22 °C for 2.5 h, after which DNA was purified with Agencourt AMPure XP beads (Beckman Coulter, Brea, CA, USA). Test PCR reactions were performed to determine the optimal number of PCR cycles necessary to generate sufficient PCR products for sequencing. The PCR reactions were performed using KAPA High Fidelity DNA Polymerase (KAPA) and Illumina PE1.0 and PE2.0 PCR primers (10 pmol each). The temperature profile was 5 min at 98 °C, followed by 9, 12, 15, and 18 cycles of 20 s at 98 °C, 15 s at 65 °C, and 20 s at 72 °C. The PCR reactions were separated on a 2% agarose gel supplemented with ethidium bromide, and the number of PCR cycles necessary to obtain a sufficient amount of DNA was determined based on visual inspection of the gels. Four preparative PCR reactions were performed for each sample. The PCR mixtures were combined, and the products were purified using Agencourt AMPure XP beads (Beckman Coulter, Brea, CA, USA). ChIP libraries were sequenced on the Illumina HiSeq 2500, resulting in 8.5–15 million 50-nt single-end reads per sample (Supplementary Table S3).

### 4.5. Hi-C Procedure

For Hi-C experiments, ~10^7^ cells per replicate were fixed in PBS containing 2% formaldehyde for 10 min with occasional mixing. The reaction was quenched by the addition of 2 M glycine to a final concentration of 125 mM. Cells were lysed in 1.5 mL isotonic buffer (50 mM Tris−HCl pH 8.0, 150 mM NaCl, 0.5% (*v*/*v*) NP-40 substitute, 1% (*v*/*v*) Triton X-100, 1 × Halt™ Protease Inhibitor Cocktail (Thermo Scientific, Waltham, MA, USA)) on ice for 15 min. The downstream steps were performed as described in [69]. Chromatin in cells after HDAC1-KD (and control) was digested with the DpnII restriction enzyme (NEB). In all other samples, chromatin was digested with the HindIII-HF restriction enzyme (NEB). Hi-C libraries were sequenced on the Illumina HiSeq 2500 and Illumina NovaSeq 6000, resulting in 26–124 million 100-nt paired-end reads per sample (Supplementary Table S3).

### 4.6. Analysis of ChIP-Seq Data

Sequencing reads were processed with the *nf-core* chipseq pipeline, using the dm3/R5 *Drosophila* genome assembly. For downstream analysis, BigWig tracks were converted to BedGraph format, and the average signal was calculated within bins matching the Hi-C map resolution used for TAD analysis (20 kb for the TSA and curcumin experiments). We generated 1-kb ChIP-seq tracks for visualization in the UCSC Genome Browser and 10-kb ChIP-seq tracks for each replicate without any normalization to assess similarity between samples. We also calculated Pearson’s correlation coefficients (Supplementary Figure S1A). Next, we separately averaged signal and input tracks across replicates and divided the mean signal by the corresponding mean input, adding a pseudocount defined as the smallest non-zero value among replicates. To plot average profiles of H4 pan-acetylation within active chromatin types, we applied *deepTools* [70] to input-normalized ChIP-seq tracks at 1-kb resolution and smoothed the obtained curves with a Savitzky–Golay filter (function savgol_filter from *SciPy*).

### 4.7. Analysis of Hi-C Data

Sequencing reads from Hi-C experiments were trimmed with *Trim Galore* (stringency 3) and processed using the *distiller-nf* pipeline (version 0.3.3) (https://doi.org/10.5281/zenodo.3350926). Reads were mapped to chromosomes 2L, 2R, 3L, 3R, 4, X, and M of the dm3/R5 *Drosophila* genome assembly (default settings, with options mapq1 ≥ 30 and mapq2 ≥ 30). After that, only chromosomes 2L, 2R, 3L, and 3R were retained. Sample similarity was assessed with *HiCRep* [71] based on the stratum-adjusted correlation coefficient (SCC), calculated for 20-kb-resolution Hi-C maps for bins separated by ≤0.5 Mb, with downsampling (Supplementary Figure S1B). Next, replicates were merged with *cooler* [72]. Because the total number of contacts in Hi-C maps for the experiments with TSA and HDAC1-KD treatments was quite different relative to their respective controls, these Hi-C matrices were downsampled with the *cooltools* [73] package to nearly the same total number of contacts within each experiment before further analysis. TAD (or super-TAD) boundaries were annotated using *Armatus* (version 2.2) [52] in 20-kb-resolution Hi-C maps (for TSA, curcumin, and DMSO treatments), or in 2-kb-resolution Hi-C maps (for HDAC1-KD and control), which were additionally preprocessed. To mask genomic regions with low mappability, Hi-C matrices were linearly interpolated along the diagonals. Contact values beyond the 1st–99th percentile range were clipped to those percentile limits. Finally, matrices were ln-transformed. *Armatus* was run for autosomes with *γ* parameters chosen by visual inspection to best match the experimental and control layouts: *γ* = 1.4 for curcumin treatment and control, *γ* = 1.4 and 1.2 for TSA treatment and control, respectively, and *γ* = 0.8 for HDAC1-KD and control. As a result, we identified 669 super-TADs upon curcumin treatment and 755 super-TADs upon control (DMSO) treatment (with 516 shared super-TADs), 931 super-TADs upon TSA treatment and 865 super-TADs upon control (DMSO) treatment (with 710 shared super-TADs), and 3814 TADs upon HDAC1-KD and 3696 TADs upon control (with 2942 shared TADs).

For downstream analysis, we focused on TADs/super-TADs with boundaries coinciding between treatment and control experiments within ±1 bin, using the coordinates of TADs/super-TADs in the control experiments as references. Average contact frequency (ACF) was calculated based on observed/expected iteratively corrected Hi-C maps as the mean value of contacts within a TAD/super-TAD (Supplementary Table S1). TADs/super-TADs were divided into 4 groups based on the quartiles of the (X − Y)/(X + Y) ratio, where X is the proportion of inactive (types 6–9) and Y is the proportion of active (types 1–5) chromatin within each TAD/super-TAD according to [51] (Supplementary Table S2). Owing to the high number of active TADs detected at 2-kb resolution in HDAC1-KD and control experiments, group A TADs were defined as having the (X − Y)/(X + Y) ratio equal to −1, and the remaining three groups were divided to contain approximately equal numbers of TADs. To analyze H4 pan-acetylation in TADs/super-TADs stratified by chromatin types, we intersected their coordinates with the normalized ChIP-seq tracks and calculated the mean signal within each TAD/super-TAD (Supplementary Table S2).

The insulation score (IS) [54] was calculated with *cooltools* using the following parameters for TSA, curcumin, and control: 10-kb-resolution Hi-C maps, a 100-kb window, with the main diagonal excluded; and for HDAC1-KD and control: 2-kb-resolution Hi-C maps, a 20-kb window, with the main diagonal excluded. Averaged IS profiles were smoothed by linear interpolation using SciPy.

Changes in interactions within A- and B-compartments were analyzed using *cooltools*. Specifically, for each 20-kb Hi-C map, a saddle plot was generated, and the difference between the experimental and control matrices was plotted.

### 4.8. Statistical Analysis

The Wilcoxon signed-rank test was applied to estimate *p*-values when comparing two distributions or to assess whether log_2_ fold-change values were symmetrically distributed around zero.

## Figures and Tables

**Figure 1:**
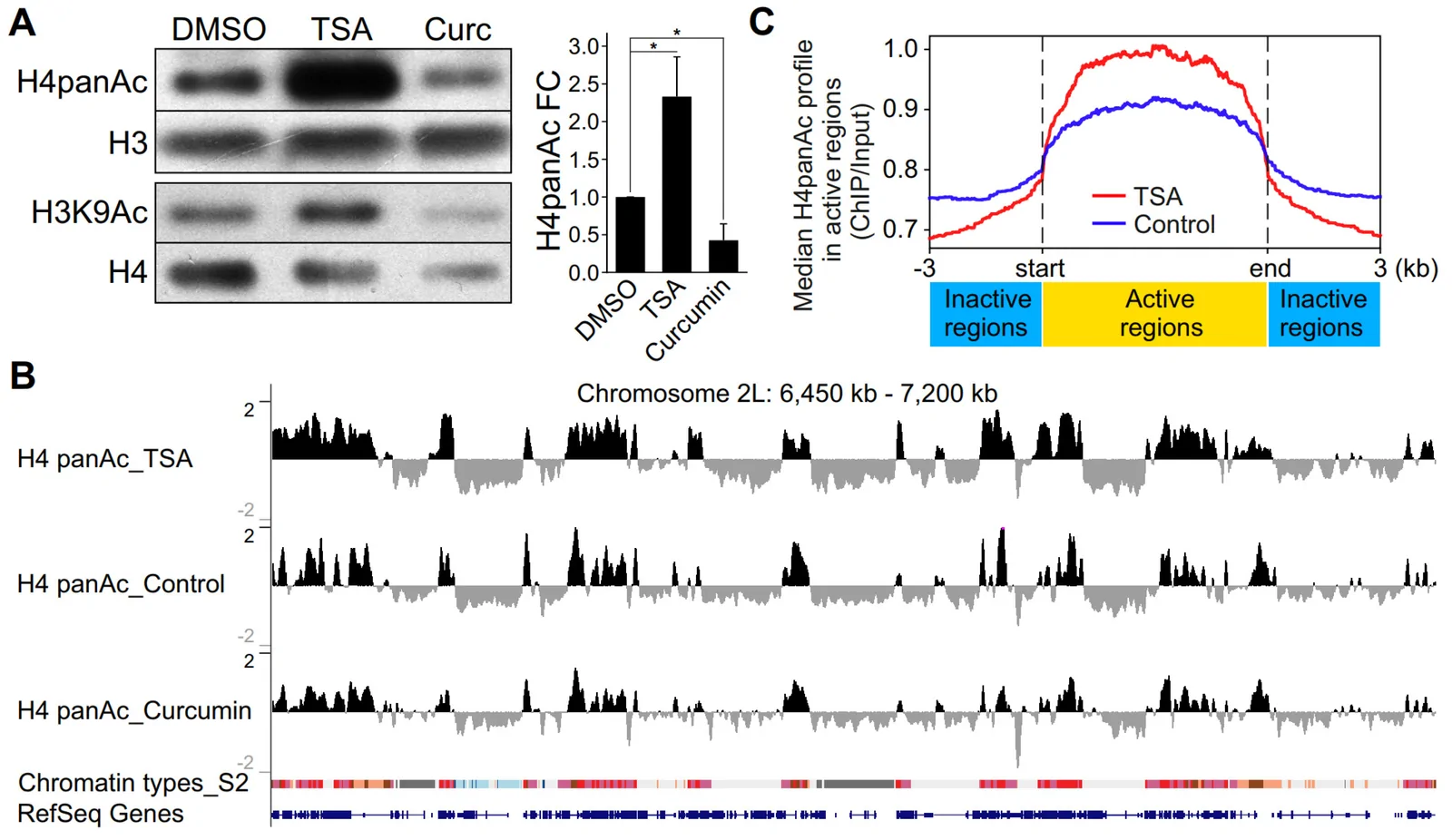
Histone acetylation changes after TSA or curcumin treatment of S2 cells. (**A**) Western blot analysis of protein extracts from S2 cells treated with TSA, curcumin, or the control (DMSO; dimethyl sulfoxide, in which both compounds were dissolved). Acetylated histones were detected using antibodies against H4panAc or H3K9Ac (**left** panel). Histones H3 and H4 were used as the loading controls. Quantification of Western blot analysis showing the fold change (FC) in histone H4 pan-acetylation relative to the control (DMSO) (**right** panel). (*) *p* < 0.05 was calculated using an unpaired *t*-test. (**B**) Screenshot from the UCSC Genome Browser showing H4panAc ChIP-seq profiles in a particular chromosomal region. Below are nine chromatin types in S2 cells [51] (active chromatin colors: red, purple, brown, coral, green; inactive chromatin colors: blue (dark and light), grey (dark and light)) and RefSeq genes. (**C**) Median H4panAc ChIP-seq profile (log_2_(FC) (ChIP/Input)) in active (chromatin types 1–5 according to [51]) or inactive (chromatin types 6–9 according to [51]) chromatin regions in control S2 cells (blue) and after treatment with TSA (red).

**Figure 2:**
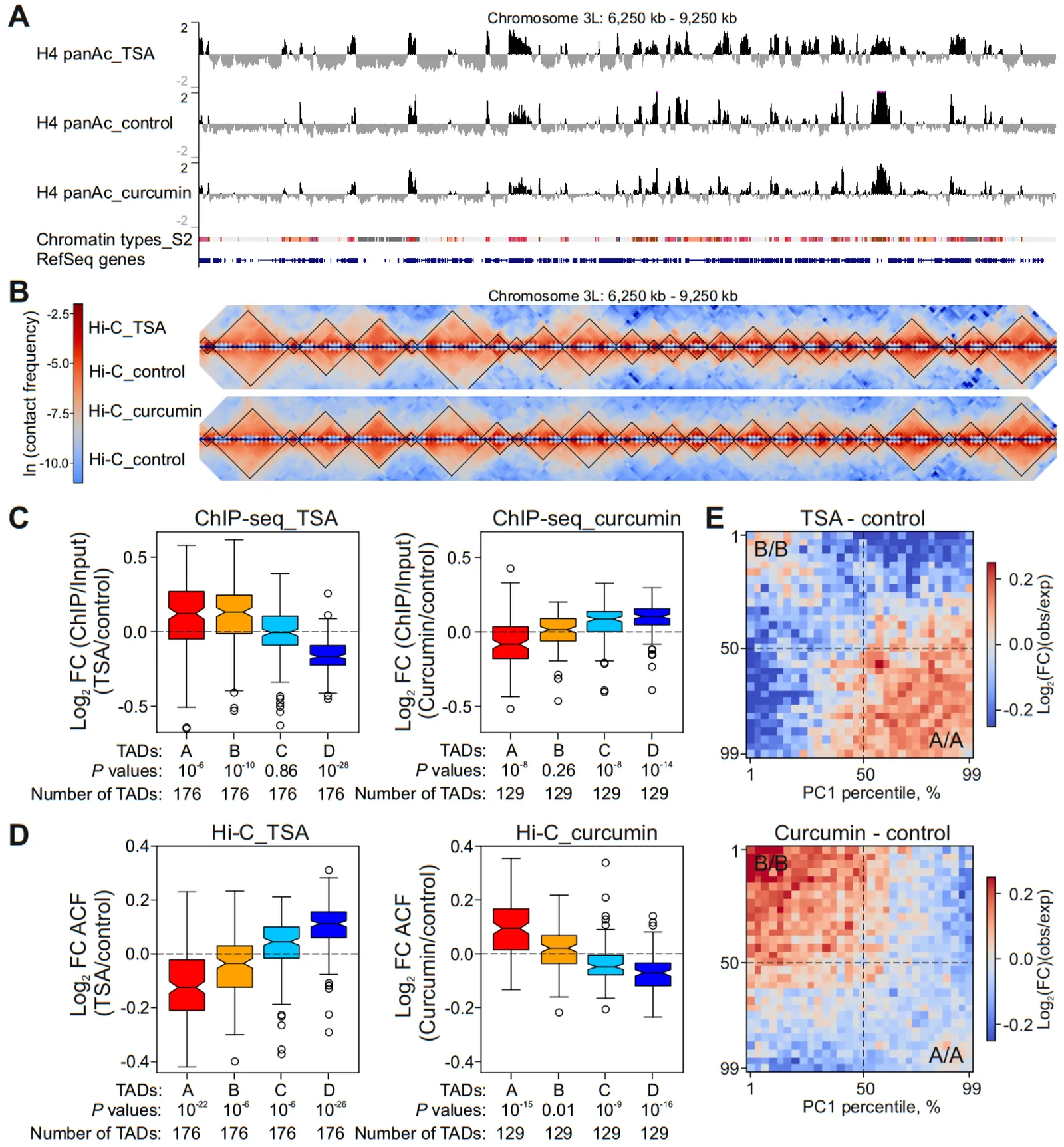
Alterations in chromatin packaging density within super-TADs inversely correlate with changes in histone acetylation. (**A**) Screenshot from the UCSC Genome Browser showing H4panAc ChIP-seq profiles in a particular region of chromosome 3L in S2 cells treated with TSA, curcumin, or DMSO (control). Below are nine chromatin types in S2 cells [51] (active chromatin colors: red, purple, brown, coral, green; inactive chromatin colors: blue (dark and light), grey (dark and light)) and RefSeq genes. (**B**) Hi-C heat map for the same region of chromosome 3L for TSA-treated S2 cells (above the diagonal) and control (DMSO-treated) S2 cells (below the diagonal) (**upper** panel), or for curcumin-treated S2 cells (above the diagonal) and control (DMSO-treated) S2 cells (below the diagonal) (**lower** panel). Super-TADs are outlined by a thin line. (**C**,**D**) Box plots showing log_2_(FC) in histone acetylation according to H4panAc ChIP-seq (**C**) or log_2_(FC) in ACF according to Hi-C (**D**) within A, B, C, and D groups of super-TADs after TSA (**left** panels) or curcumin (**right** panels) treatment of S2 cells relative to control S2 cells (treated with DMSO). *p*-values for non-symmetric distribution around zero in (**C**,**D**) were calculated using the Wilcoxon test. (**E**) Saddle plots showing the subtraction of intra-chromosomal contact enrichment values across all chromosomes in the TSA Hi-C map relative to the control (**upper** panel), or in the curcumin Hi-C map relative to the control (**lower** panel).

**Figure 3:**
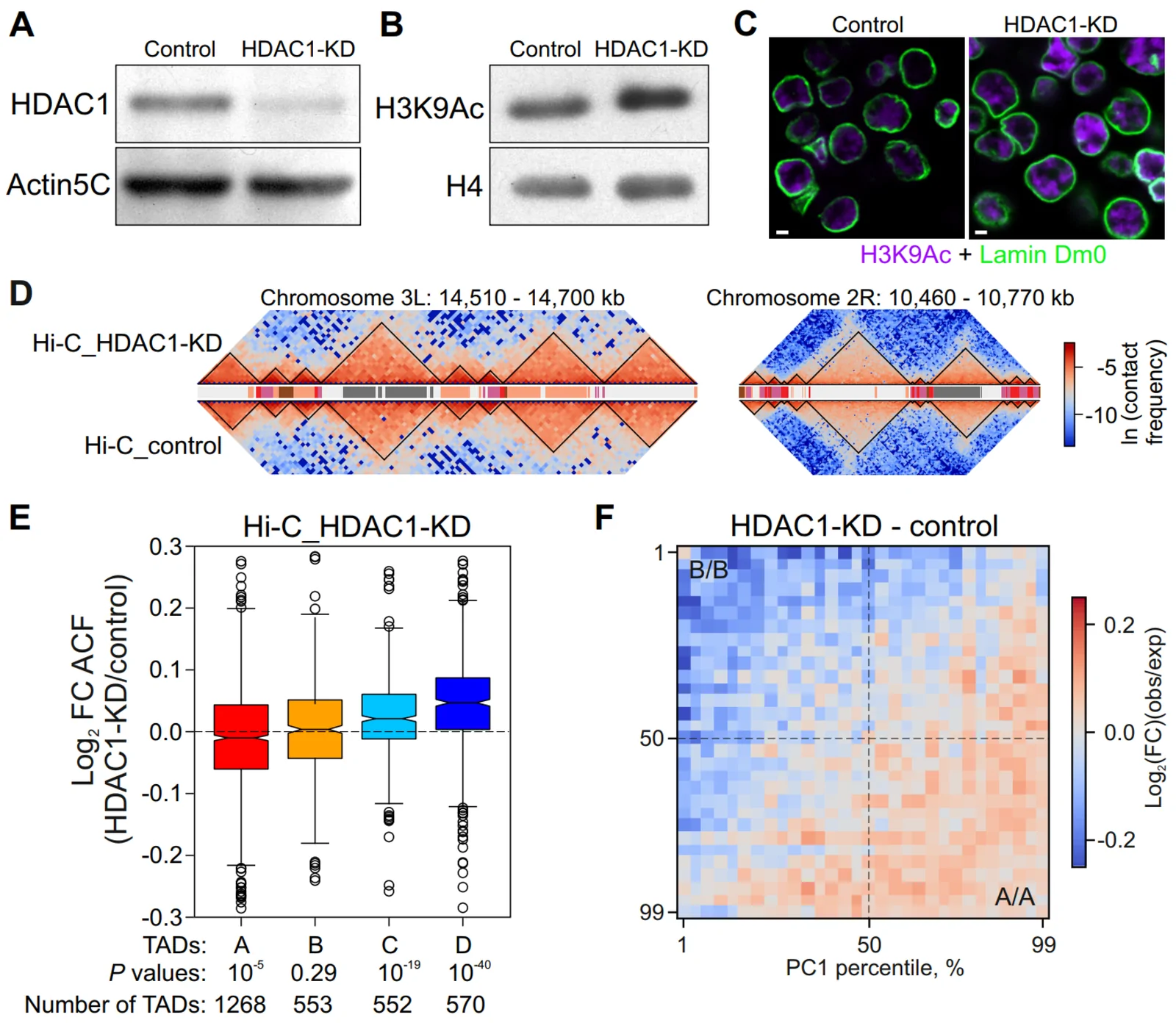
Alterations in chromatin packaging density within TADs inversely correlate with changes in histone acetylation. (**A**,**B**) Western blot analysis of protein extracts from control and HDAC1-KD S2 cells. Blots were stained with antibodies against HDAC1 and Actin5C (**A**) or against H3K9Ac and histone H4 (**B**). (**C**) Immunostaining of control and HDAC1-KD S2 cells with anti-H3K9Ac (violet) and anti-lamin Dm0 (green) antibodies. Scale bar, 1 µm. (**D**) Hi-C heat maps for representative regions of chromosome 3L (**left**) and 2R (**right**) for HDAC1-KD S2 cells (above the diagonal) and control S2 cells (below the diagonal). TADs are outlined by a thin line. The track corresponding to nine chromatin types in S2 cells [51] (active chromatin colors: red, purple, brown, coral, green; inactive chromatin colors: blue (dark and light), grey (dark and light)) is shown between the Hi-C maps. (**E**) Box plots showing log_2_(FC) in ACF according to Hi-C within A, B, C, and D groups of TADs in HDAC1-KD S2 cells relative to control S2 cells. *p*-values for a non-symmetric distribution around zero were calculated using the Wilcoxon test. (**F**) Saddle plot showing the subtraction of intra-chromosomal contact enrichment values across all chromosomes in the HDAC1-KD Hi-C map relative to the control.

**Figure 4:**
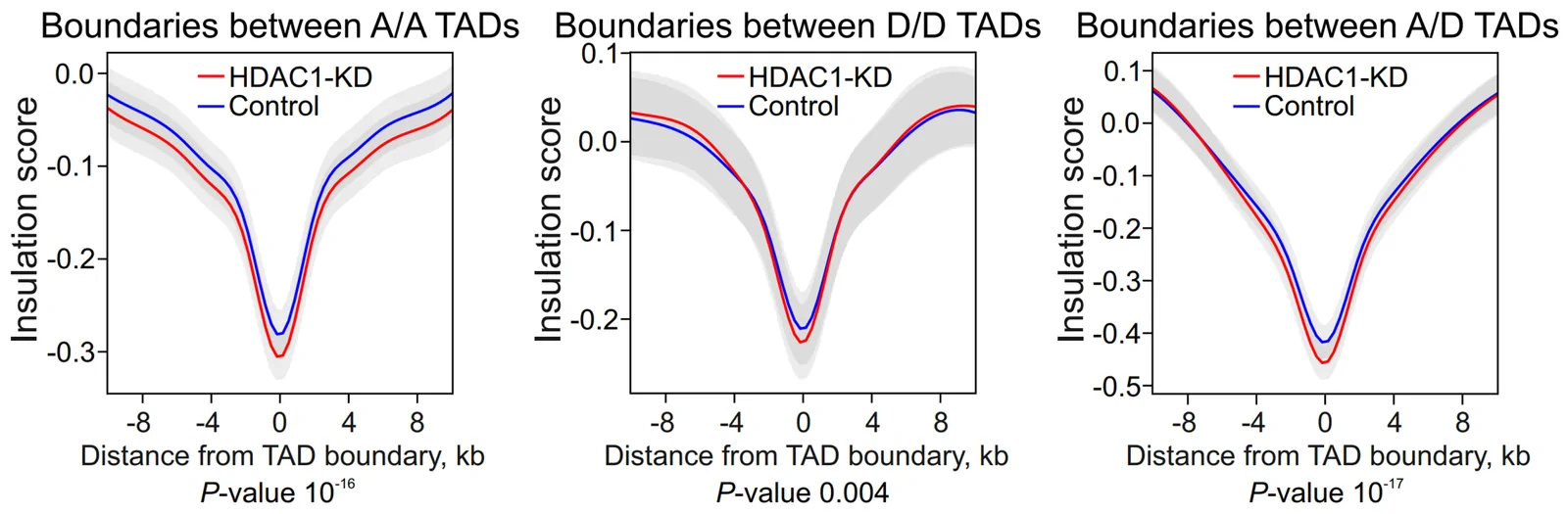
Histone acetylation increases the degree of insulation between TADs of various groups. Average insulation score in HDAC1-KD (red line) and control (blue line) cells between adjacent TADs of group A (**left panel**), group D (**middle panel**), or groups A/D (**right panel**). *p*-values were calculated using the Wilcoxon test.

## Data Availability

Raw and processed ChIP-seq and Hi-C data were deposited in the NCBI Gene Expression Omnibus (GEO) under the accession numbers GSE334863 for ChIP-seq and GSE334862 and GSE345637 for Hi-C.

## Supplementary Materials

The following supporting information can be downloaded at https://www.mdpi.com/article/10.3390/ijms27198638/s1.

## References

[1] Chen Y.C., Koutelou E., Dent S.Y.R. (2022). Now open: Evolving insights to the roles of lysine acetylation in chromatin organization and function. Mol. Cell.

[2] Josling G.A., Selvarajah S.A., Petter M., Duffy M.F. (2012). The role of bromodomain proteins in regulating gene expression. Genes.

[3] Qiu X., Xiao X., Li N., Li Y. (2017). Histone deacetylases inhibitors (HDACis) as novel therapeutic application in various clinical diseases. Prog. Neuropsychopharmacol. Biol. Psychiatry.

[4] Eberharter A., Becker P.B. (2002). Histone acetylation: A switch between repressive and permissive chromatin. Second in review series on chromatin dynamics. EMBO Rep..

[5] Shevelyov Y.Y., Nurminsky D.I. (2012). The nuclear lamina as a gene-silencing hub. Curr. Issues Mol. Biol..

[6] Lieberman-Aiden E., van Berkum N.L., Williams L., Imakaev M., Ragoczy T., Telling A., Amit I., Lajoie B.R., Sabo P.J., Dorschner M.O. (2009). Comprehensive mapping of long-range interactions reveals folding principles of the human genome. Science.

[7] Dixon J.R., Selvaraj S., Yue F., Kim A., Li Y., Shen Y., Hu M., Liu J.S., Ren B. (2012). Topological domains in mammalian genomes identified by analysis of chromatin interactions. Nature.

[8] Hou C., Li L., Qin Z.S., Corces V.G. (2012). Gene density, transcription, and insulators contribute to the partition of the *Drosophila* genome into physical domains. Mol. Cell.

[9] Nora E.P., Lajoie B.R., Schulz E.G., Giorgetti L., Okamoto I., Servant N., Piolot T., van Berkum N.L., Meisig J., Sedat J. (2012). Spatial partitioning of the regulatory landscape of the X-inactivation centre. Nature.

[10] Sexton T., Yaffe E., Kenigsberg E., Bantignies F., Leblanc B., Hoichman M., Parrinello H., Tanay A., Cavalli G. (2012). Three-dimensional folding and functional organization principles of the *Drosophila* genome. Cell.

[11] Sanborn A.L., Rao S.S., Huang S.C., Durand N.C., Huntley M.H., Jewett A.I., Bochkov I.D., Chinnappan D., Cutkosky A., Li J. (2015). Chromatin extrusion explains key features of loop and domain formation in wild-type and engineered genomes. Proc. Natl. Acad. Sci. USA.

[12] Fudenberg G., Imakaev M., Lu C., Goloborodko A., Abdennur N., Mirny L.A. (2016). Formation of chromosomal domains by loop extrusion. Cell Rep..

[13] Rao S.S.P., Huang S.C., Glenn St Hilaire B., Engreitz J.M., Perez E.M., Kieffer-Kwon K.R., Sanborn A.L., Johnstone S.E., Bascom G.D., Bochkov I.D. (2017). Cohesin loss eliminates all loop domains. Cell.

[14] Rowley M.J., Nichols M.H., Lyu X., Ando-Kuri M., Rivera I.S.M., Hermetz K., Wang P., Ruan Y., Corces V.G. (2017). Evolutionarily conserved principles predict 3D chromatin organization. Mol. Cell.

[15] Schwarzer W., Abdennur N., Goloborodko A., Pekowska A., Fudenberg G., Loe-Mie Y., Fonseca N.A., Huber W., Haering C.H., Mirny L. (2017). Two independent modes of chromatin organization revealed by cohesin removal. Nature.

[16] Nora E.P., Goloborodko A., Valton A.L., Gibcus J.H., Uebersohn A., Abdennur N., Dekker J., Mirny L.A., Bruneau B.G. (2017). Targeted degradation of CTCF decouples local insulation of chromosome domains from genomic compartmentalization. Cell.

[17] Nichols M.H., Corces V.G. (2021). Principles of 3D compartmentalization of the human genome. Cell Rep..

[18] Rowley M.J., Corces V.G. (2018). Organizational principles of 3D genome architecture. Nat. Rev. Genet..

[19] Ulianov S.V., Khrameeva E.E., Gavrilov A.A., Flyamer I.M., Kos P., Mikhaleva E.A., Penin A.A., Logacheva M.D., Imakaev M.V., Chertovich A. (2016). Active chromatin and transcription play a key role in chromosome partitioning into topologically associating domains. Genome Res..

[20] Ramírez F., Bhardwaj V., Arrigoni L., Lam K.C., Grüning B.A., Villaveces J., Habermann B., Akhtar A., Manke T. (2018). High-resolution TADs reveal DNA sequences underlying genome organization in flies. Nat. Commun..

[21] Wang Q., Sun Q., Czajkowsky D.M., Shao Z. (2018). Sub-kb Hi-C in *D. melanogaster* reveals conserved characteristics of TADs between insect and mammalian cells. Nat. Commun..

[22] Zhang H., Shi Z., Banigan E.J., Kim Y., Yu H., Bai X.C., Finkelstein I.J. (2023). CTCF and R-loops are boundaries of cohesin-mediated DNA looping. Mol. Cell.

[23] Jost D., Carrivain P., Cavalli G., Vaillant C. (2014). Modeling epigenome folding: Formation and dynamics of topologically associated chromatin domains. Nucleic Acids Res..

[24] Falk M., Feodorova Y., Naumova N., Imakaev M., Lajoie B.R., Leonhardt H., Joffe B., Dekker J., Fudenberg G., Solovei I. (2019). Heterochromatin drives compartmentalization of inverted and conventional nuclei. Nature.

[25] Wang L., Gao Y., Zheng X., Liu C., Dong S., Li R., Zhang G., Wei Y., Qu H., Li Y. (2019). Histone modifications regulate chromatin compartmentalization by contributing to a phase separation mechanism. Mol. Cell.

[26] Lee Y.C.G., Ogiyama Y., Martins N.M.C., Beliveau B.J., Acevedo D., Wu C.T., Cavalli G., Karpen G.H. (2020). Pericentromeric heterochromatin is hierarchically organized and spatially contacts H3K9me2 islands in euchromatin. PLoS Genet..

[27] Luger K., Mäder A.W., Richmond R.K., Sargent D.F., Richmond T.J. (1997). Crystal structure of the nucleosome core particle at 2.8 A resolution. Nature.

[28] Sinha D., Shogren-Knaak M.A. (2010). Role of direct interactions between the histone H4 tail and the H2A core in long range nucleosome contacts. J. Biol. Chem..

[29] Shogren-Knaak M., Ishii H., Sun J.M., Pazin M.J., Davie J.R., Peterson C.L. (2006). Histone H4-K16 acetylation controls chromatin structure and protein interactions. Science.

[30] Allahverdi A., Yang R., Korolev N., Fan Y., Davey C.A., Liu C.F., Nordenskiöld L. (2011). The effects of histone H4 tail acetylations on cation-induced chromatin folding and self-association. Nucleic Acids Res..

[31] Zhang R., Erler J., Langowski J. (2017). Histone acetylation regulates chromatin accessibility: Role of H4K16 in inter-nucleosome interaction. Biophys. J..

[32] Fukai Y.T., Kujirai T., Wakamori M., Kanamura S., Yamauchi L., Zeraati S., Morita S., Tanegashima C., Kadota M., Shirouzu M. (2025). Gene-scale in vitro reconstitution reveals histone acetylation directly controls chromatin architecture. Sci. Adv..

[33] Larson A.G., Elnatan D., Keenen M.M., Trnka M.J., Johnston J.B., Burlingame A.L., Agard D.A., Redding S., Narlikar G.J. (2017). Liquid droplet formation by HP1α suggests a role for phase separation in heterochromatin. Nature.

[34] Strom A.R., Emelyanov A.V., Mir M., Fyodorov D.V., Darzacq X., Karpen G.H. (2017). Phase separation drives heterochromatin domain formation. Nature.

[35] Gibson B.A., Doolittle L.K., Schneider M.W.G., Jensen L.E., Gamarra N., Henry L., Gerlich D.W., Redding S., Rosen M.K. (2019). Organization of chromatin by intrinsic and regulated phase separation. Cell.

[36] Tóth K.F., Knoch T.A., Wachsmuth M., Frank-Stöhr M., Stöhr M., Bacher C.P., Müller G., Rippe K. (2004). Trichostatin A-induced histone acetylation causes decondensation of interphase chromatin. J. Cell Sci..

[37] Boettiger A.N., Bintu B., Moffitt J.R., Wang S., Beliveau B.J., Fudenberg G., Imakaev M., Mirny L.A., Wu C.T., Zhuang X. (2016). Super-resolution imaging reveals distinct chromatin folding for different epigenetic states. Nature.

[38] Nozaki T., Imai R., Tanbo M., Nagashima R., Tamura S., Tani T., Joti Y., Tomita M., Hibino K., Kanemaki M.T. (2017). Dynamic organization of chromatin domains revealed by super-resolution live-cell imaging. Mol. Cell.

[39] Xu J., Ma H., Jin J., Uttam S., Fu R., Huang Y., Liu Y. (2018). Super-resolution imaging of higher-order chromatin structures at different epigenomic states in single mammalian cells. Cell Rep..

[40] Otterstrom J., Castells-Garcia A., Vicario C., Gomez-Garcia P.A., Cosma M.P., Lakadamyali M. (2019). Super-resolution microscopy reveals how histone tail acetylation affects DNA compaction within nucleosomes in vivo. Nucleic Acids Res..

[41] Foglietti C., Filocamo G., Cundari E., De Rinaldis E., Lahm A., Cortese R., Steinkühler C. (2006). Dissecting the biological functions of *Drosophila* histone deacetylases by RNA interference and transcriptional profiling. J. Biol. Chem..

[42] Finnin M.S., Donigian J.R., Cohen A., Richon V.M., Rifkind R.A., Marks P.A., Breslow R., Pavletich N.P. (1999). Structures of a histone deacetylase homologue bound to the TSA and SAHA inhibitors. Nature.

[43] Balasubramanyam K., Varier R.A., Altaf M., Swaminathan V., Siddappa N.B., Ranga U., Kundu T.K. (2004). Curcumin, a novel p300/CREB-binding protein-specific inhibitor of acetyltransferase, represses the acetylation of histone/nonhistone proteins and histone acetyltransferase-dependent chromatin transcription. J. Biol. Chem..

[44] Morimoto T., Sunagawa Y., Kawamura T., Takaya T., Wada H., Nagasawa A., Komeda M., Fujita M., Shimatsu A., Kita T. (2008). The dietary compound curcumin inhibits p300 histone acetyltransferase activity and prevents heart failure in rats. J. Clin. Investig..

[45] Marcu M.G., Jung Y.J., Lee S., Chung E.J., Lee M.J., Trepel J., Neckers L. (2006). Curcumin is an inhibitor of p300 histone acetylatransferase. Med. Chem..

[46] Liu H.L., Chen Y., Cui G.H., Zhou J.F. (2005). Curcumin, a potent anti-tumor reagent, is a novel histone deacetylase inhibitor regulating B-NHL cell line Raji proliferation. Acta Pharmacol. Sin..

[47] Chen Y., Shu W., Chen W., Wu Q., Liu H., Cui G. (2007). Curcumin, both histone deacetylase and p300/CBP-specific inhibitor, represses the activity of nuclear factor kappa B and Notch 1 in Raji cells. Basic. Clin. Pharmacol. Toxicol..

[48] Lee S.J., Krauthauser C., Maduskuie V., Fawcett P.T., Olson J.M., Rajasekaran S.A. (2011). Curcumin-induced HDAC inhibition and attenuation of medulloblastoma growth in vitro and in vivo. BMC Cancer.

[49] Bora-Tatar G., Dayangaç-Erden D., Demir A.S., Dalkara S., Yelekçi K., Erdem-Yurter H. (2009). Molecular modifications on carboxylic acid derivatives as potent histone deacetylase inhibitors: Activity and docking studies. Bioorg. Med. Chem..

[50] Anandaradje A., Kalita B., Coumar M.S., Selvarajan S. (2024). Molecular docking of curcumin and curcuminoids as human Zn^+^ dependent histone deacetylase (HDAC) enzyme inhibitors. In Silico Pharmacol..

[51] Kharchenko P.V., Alekseyenko A.A., Schwartz Y.B., Minoda A., Riddle N.C., Ernst J., Sabo P.J., Larschan E., Gorchakov A.A., Gu T. (2011). Comprehensive analysis of the chromatin landscape in *Drosophila melanogaster*. Nature.

[52] Filippova D., Patro R., Duggal G., Kingsford C. (2014). Identification of alternative topological domains in chromatin. Algorithms Mol. Biol..

[53] Zhang Y., Malone J.H., Powell S.K., Periwal V., Spana E., Macalpine D.M., Oliver B. (2010). Expression in aneuploid *Drosophila* S2 cells. PLoS Biol..

[54] Crane E., Bian Q., McCord R.P., Lajoie B.R., Wheeler B.S., Ralston E.J., Uzawa S., Dekker J., Meyer B.J. (2015). Condensin-driven remodelling of X chromosome topology during dosage compensation. Nature.

[55] Lopez-Atalaya J.P., Ito S., Valor L.M., Benito E., Barco A. (2013). Genomic targets, and histone acetylation and gene expression profiling of neural HDAC inhibition. Nucleic Acids Res..

[56] Paldi F., Szalay M.F., Dufau S., Di Stefano M., Reboul H., Jost D., Bantignies F., Cavalli G. (2026). Transient histone deacetylase inhibition induces cellular memory of gene expression and 3D genome folding. Nat. Genet..

[57] Wang Z., Zang C., Cui K., Schones D.E., Barski A., Peng W., Zhao K. (2009). Genome-wide mapping of HATs and HDACs reveals distinct functions in active and inactive genes. Cell.

[58] Peserico A., Simone C. (2011). Physical and functional HAT/HDAC interplay regulates protein acetylation balance. J. Biomed. Biotechnol..

[59] Ricci M.A., Manzo C., García-Parajo M.F., Lakadamyali M., Cosma M.P. (2015). Chromatin fibers are formed by heterogeneous groups of nucleosomes in vivo. Cell.

[60] Smith R.G., Fu Y., Schiela K.L., Dautle M., Williams R., Wilson H.M., Azadegan C., Whetstine J.R., Dekker J., Liu Y. (2025). Histone acetylation differentially modulates CTCF-CTCF loops and intra-TAD interactions. bioRxiv.

[61] Szabo Q., Bantignies F., Cavalli G. (2019). Principles of genome folding into topologically associating domains. Sci. Adv..

[62] Schneider I. (1972). Cell lines derived from late embryonic stages of *Drosophila melanogaster*. J. Embryol. Exp. Morphol..

[63] Pindyurin A.V., Ilyin A.A., Ivankin A.V., Tselebrovsky M.V., Nenasheva V.V., Mikhaleva E.A., Pagie L., van Steensel B., Shevelyov Y.Y. (2018). The large fraction of heterochromatin in *Drosophila* neurons is bound by both B-type lamin and HP1a. Epigenet. Chromatin.

[64] Ulianov S.V., Doronin S.A., Khrameeva E.E., Kos P.I., Luzhin A.V., Starikov S.S., Galitsyna A.A., Nenasheva V.V., Ilyin A.A., Flyamer I.M. (2019). Nuclear lamina integrity is required for proper spatial organization of chromatin in *Drosophila*. Nat. Commun..

[65] Karoutas A., Szymanski W., Rausch T., Guhathakurta S., Rog-Zielinska E.A., Peyronnet R., Seyfferth J., Chen H.R., de Leeuw R., Herquel B. (2019). The NSL complex maintains nuclear architecture stability via lamin A/C acetylation. Nat. Cell Biol..

[66] Yudaev P., Aleksandrova Y., Neganova M. (2025). Recent insights into the creation of histone deacetylase inhibitors for the treatment of human diseases. Int. J. Mol. Sci..

[67] Shevelyov Y.Y., Lavrov S.A., Mikhaylova L.M., Nurminsky I.D., Kulathinal R.J., Egorova K.S., Rozovsky Y.M., Nurminsky D.I. (2009). The B-type lamin is required for somatic repression of testis-specific gene clusters. Proc. Natl. Acad. Sci. USA.

[68] Clemens J.C., Worby C.A., Simonson-Leff N., Muda M., Maehama T., Hemmings B.A., Dixon J.E. (2000). Use of double-stranded RNA interference in *Drosophila* cell lines to dissect signal transduction pathways. Proc. Natl. Acad. Sci. USA.

[69] Ilyin A.A., Ryazansky S.S., Doronin S.A., Olenkina O.M., Mikhaleva E.A., Yakushev E.Y., Abramov Y.A., Belyakin S.N., Ivankin A.V., Pindyurin A.V. (2017). Piwi interacts with chromatin at nuclear pores and promiscuously binds nuclear transcripts in *Drosophila* ovarian somatic cells. Nucleic Acids Res..

[70] Ramírez F., Ryan D.P., Grüning B., Bhardwaj V., Kilpert F., Richter A.S., Heyne S., Dündar F., Manke T. (2016). deepTools2: A next generation web server for deep-sequencing data analysis. Nucleic Acids Res..

[71] Yang T., Zhang F., Yardımcı G.G., Song F., Hardison R.C., Noble W.S., Yue F., Li Q. (2017). HiCRep: Assessing the reproducibility of Hi-C data using a stratum-adjusted correlation coefficient. Genome Res..

[72] Abdennur N., Mirny L.A. (2020). Cooler: Scalable storage for Hi-C data and other genomically labeled arrays. Bioinformatics.

[73] Abdennur N., Abraham S., Fudenberg G., Flyamer I.M., Galitsyna A.A., Goloborodko A., Imakaev M., Oksuz B.A., Venev S.V., Open2C (2024). Cooltools: Enabling high-resolution Hi-C analysis in Python. PLoS Comput. Biol..

