# Supplementary material for: Influence of Histone Acetylation on Chromatin 3D Organization in Drosophila S2 Cells
Source: Int J Mol Sci. 2026 Sep 27;27(19):8638. doi: 10.3390/ijms27198638 (PMC13664830; doi:10.3390/ijms27198638)
Supplement: Supplementary file 1 [file ijms-27-08638-s001.zip › Supplementary Figure S1.pdf]

A

Pearson correlation coefficients between ChIP-seq samples

	TSA1	TSA2	Curcumin1	Curcumin2	DMSO1	DMSO2	TSA_input1	TSA_input2	Curcumin_input1	Curcumin_input2	DMSO_input1	DMSO_input2
TSA1		0,99	0,89	0,88	0,87	0,88	0,83	0,74	0,74	0,71	0,78	0,72
TSA2	0,99		0,90	0,89	0,87	0,89	0,85	0,79	0,77	0,75	0,82	0,76
Curcumin1	0,89	0,90		1,00	0,95	0,96	0,85	0,79	0,81	0,79	0,84	0,78
Curcumin2	0,88	0,89	1,00		0,95	0,96	0,85	0,80	0,81	0,79	0,84	0,79
DMSO1	0,87	0,87	0,95	0,95		0,98	0,77	0,71	0,72	0,70	0,75	0,70
DMSO2	0,88	0,89	0,96	0,96	0,98		0,81	0,74	0,75	0,74	0,79	0,74
TSA_input1	0,83	0,85	0,85	0,85	0,77	0,81		0,96	0,96	0,96	0,98	0,96
TSA_input2	0,74	0,79	0,79	0,80	0,71	0,74	0,96		0,97	0,97	0,99	0,99
Curcumin_input1	0,74	0,77	0,81	0,81	0,72	0,75	0,96	0,97		0,99	0,98	0,98
Curcumin_input2	0,71	0,75	0,79	0,79	0,70	0,74	0,96	0,97	0,99		0,97	0,98
DMSO_input1	0,78	0,82	0,84	0,84	0,75	0,79	0,98	0,99	0,98	0,97		0,99
DMSO_input2	0,72	0,76	0,78	0,79	0,70	0,74	0,96	0,99	0,98	0,98	0,99	

B

Stratum-adjusted correlation coefficients between Hi-C samples

	HDAC1-KD1	HDAC1-KD2	dsLacZ1	dsLacZ2	TSA1	TSA2	DMSO1_to_TSA	DMSO2_to_TSA	Curcumin1	Curcumin2	DMSO1_to_curc	DMSO2_to_curc
HDAC1-KD1		0,98	0,98	0,98	0,78	0,75	0,71	0,75	0,73	0,72	0,75	0,74
HDAC1-KD2	0,98		0,98	0,97	0,78	0,75	0,70	0,74	0,74	0,72	0,75	0,75
dsLacZ1	0,98	0,98		0,99	0,77	0,75	0,71	0,74	0,74	0,73	0,75	0,75
dsLacZ2	0,98	0,97	0,99		0,77	0,74	0,71	0,74	0,73	0,73	0,75	0,74
TSA1	0,78	0,78	0,77	0,77		0,95	0,92	0,95	0,92	0,91	0,93	0,92
TSA2	0,75	0,75	0,75	0,74	0,95		0,95	0,94	0,92	0,91	0,91	0,91
DMSO1_to_TSA	0,71	0,70	0,71	0,71	0,92	0,95		0,95	0,93	0,93	0,92	0,92
DMSO2_to_TSA	0,75	0,74	0,74	0,74	0,95	0,94	0,95		0,94	0,94	0,95	0,95
Curcumin1	0,73	0,74	0,74	0,73	0,92	0,92	0,93	0,94		0,98	0,96	0,96
Curcumin2	0,72	0,72	0,73	0,73	0,91	0,91	0,93	0,94	0,98		0,96	0,96
DMSO1_to_curc	0,75	0,75	0,75	0,75	0,93	0,91	0,92	0,95	0,96	0,96		0,98
DMSO2_to_curc	0,74	0,75	0,75	0,74	0,92	0,91	0,92	0,95	0,96	0,96	0,98	

Supplementary Figure S1. ChIP-seq and Hi-C replicates are highly correlated. (**A**)Pearson correlation coefficients between ChIP-seq samples. (**B**) Stratum-adjusted correlation coefficients between Hi-C samples.
